# "The Hospital" Nursing Mirror

**Published:** 1898-03-12

**Authors:** 


### "The Hospital" Nursing Mirror.

Being the Nursing Section of "The Hospital."

[Contributions for this Section of "The Hospital" should be addressed to the Editor, The Hospital, 28 & 29, Southampton Street, Strand, London, W.C., and should have the word "Nursing" plainly written in left-hand top corner of the envelope.]

### News from the Nursing World.

### THE LADIES' COMMITTEE OF THE ORTHOPÆDIC.

The Orthopædic Hospital is most fortunate in its Ladies' Committee. The Duchess of Marlborough is president, and often visits it, and spends a good deal of time chatting with the small patients, besides taking an active part in the work. She is a favourite with everyone, nurses and patients alike. Another member who has rendered the hospital good service is Mrs. Cooper. She designed a neat little calendar for the purse, adorned on the front cover by a photo of a little fellow suffering from spinal disease. She had a thousand printed at her own expense and distributed, securing as a result several subscribers. The photo is the more interesting as it was taken by a sister who has been at the hospital for ten years, and the subject made a complete recovery. The Ladies' Committee have also been generous in the matter of lockers, and have presented six handsome ones of oak. Three others have been bought by the subscriptions gathered amongst the out-patients by the matron. Much has been accomplished in every way during the last ten years, and yet everyone seems desirous of continued improvements.

### POUND DAY AT THE CHILDREN'S HOSPITAL, BRIGHTON.

The birthday of the Princess of Wales has been set apart by the committee of the Children's Hospital at Brighton as Pound-day. Pound-day is by no means a new institution in some charities, but it is a popular one. The idea is that friends each send a pound of some useful article, or the money to buy it with. In the case of this hospital the result of the first pound-day was £33 in money and £26 in kind. As the Princess of Wales is a great favourite, this method of keeping her birthday is sure to result in one or more handsome gifts, such, for instance, as that of Mr. Charles Chetwode Baily, who sent a donation of £100 to name a cot in honour of H R. Highness' visit on March 10th, 1896. We commend the idea as likely to prove attractive. Why should not loyal admirers of the reigning house have the opportunity of paying a graceful compliment to its individual members; and, moreover, one that is sure to be appreciated, whilst at the same time encouraging their local charities?

### MISS PHILLIPA HICKS.

We regret that Miss Phillipa Hicks has found it desirable to relinquish her charming house at West Ilsley, near Newbury, which she has carried on as a home for invalids since her retirement from her post at the Nurses' Co-operation. Miss Hicks finds that the distance from London is rather too great for her purpose. In other respects the Home was admirably adapted for the purpose to which she devoted it, and the appointments and arrangements were delightfully carried out for the comfort of the inmates, which those who know Miss Hicks' abilities will understand would be the case. We hope that Miss Hicks will take a much-needed rest, free from anxiety or responsibility for some time to come. Her self-sacrificing and arduous work on behalf of the Nurses' Co-operation has so tried her strength as to make this most necessary.

### THE MEDICO-PSYCHOLOGICAL EXAMINATION.

Some of our readers will doubtless like to be reminded that the next examination of the Medico-Psychological Society for the nursing of and attending on the insane will be held on Monday, May 2nd, 1898; April 4th will be the last day upon which, according to the rules, candidates can enter their names for it. They should obtain a schedule from the Registrar (Dr. Spence), Burwood Asylum, near Lichfield, to be filled up, signed, and returned at least four weeks before the date fixed for the examination.

### IRISH WORKHOUSE ASSOCIATION.

The Irish Workhouse Association has had a busy and successful year, and has an energetic programme in hand for the ensuing one. Lord Monteagle presided at the annual meeting which was held at the Mansion House, Dublin, and stated that the association numbered 176 members, and had a balance in hand. His Lordship said the Local Government Board order for the abolition of pauper nurses in the workhouses was the result of the representations of the association, and added that although the order had been in operation for five months its results were as yet small, and some time must elapse before the authorities could adjust themselves to the new state of things. He recommended reformers to direct their energies to two points, namely, the inspection of the children by the State, and improving the local supervision of the unions. As yet there are no lady inspectors of children as in England, and the local supervision is very defective. A public meeting was afterwards held, at which the Lord Mayor took the chair. Lord Monteagle, in asking him to preside, remarked that they might congratulate themselves on having brought the Government to the point of paying half the workhouse nurses' salaries. The Lord Mayor reviewed the working of the Poor Law in Ireland since its establishment, and condemned it. He wishes to see children brought up without the stigma that attaches to the pauper, and removed from the demoralising influence exercised by paupers over those that associate with them. He desires that old couples should be allowed to live together, and administer to each other's comforts, and to see the strong taught to earn an honest living. These are excellent aims to work for, and although the results must fall short, owing to the unpromising material to be dealt with, yet much may be done to minimise the evils.

### HOMES FOR WORKING GIRLS IN LONDON.

Prevention is always better than cure, therefore the appeal now being made to enlarge Garfield House, Fitzroy Square, by 50 beds in order to afford extra accommodation for respectable young girls earning their living in London, should meet with sympathetic response. There are now nine homes for working girls, and everyone under 21 years of age and bearing a good character is eligible. The work has none of the excitement of so-called rescue work, but it is even more valuable. Certainly it is not a small thing to have provided happy homes for 26,000 young girls, which this society has done, probably ensuring to a large proportion that happiness which is due to right living. Once a woman crosses the rubicon she becomes an outcast. Clothe the fallen with the largest garb of charity and pity, win back her erring feet to the paths of righteousness, make her days bright with human kindness, yet can she never forget the inward shame. Let all who love women, therefore, strengthen the hands of those who labour that girls may so live that in the days to come they may have nothing to forget. Eight hundred pounds for 50 girls to have a home is the plea set forth. Subscriptions may be sent to Mr. John Shrimpton, 3, Victoria Street, Westminster.

### LIFE PENSIONS FOR THE NURSES OF THE ROYAL BERKSHIRE HOSPITAL.

It was announced at the annual meeting of the Royal Berkshire Hospital that arrangements had been made to induce fully-qualified nurses to join the Royal National Pension Fund. This step is the result of careful inquiry. The hospital has for several years had a private scheme of its own to provide nurses with a definite sum on retirement, which has been useful in many ways; but the committee appointed to inquire into the matter consider that, as the Royal National Pension Fund is established on firm financial basis, and holds out advantages superior to those possible to any private scheme, their nurses should be encouraged to become policy-holders for pensions in it. There is an enormous satisfaction in the thought that, come weal or woe, after the age of 55 or 60, as the case may be, the toiler may, to speak figuratively, spend the sabbath of her days beside the well she has digged, and eat of the fruit of her own vine. Unfortunately, there are many nurses who forget that the well has to be dug and the vine planted in the heyday of youth and strength; or, in other words, that premiums must be paid now if age is to be blessed with independence and plenty. This is the second commission within the last few months inquiring into the best and safest agency for securing adequate support to the nurse in age that has emphasised the fact that, taking all things into consideration, no other society offers the benefits equal to those assured by the Royal National Pension Fund.

### ADDITIONS TO THE NEW HOSPITAL FOR WOMEN.

The nurses' quarters at the New Hospital for Women, Euston Road, are very comfortable. Some of the bedrooms contain one bed, but others two or (in a few cases only) three. Even these rooms, however, are divided into cubicles by curtains, so that there is not much to find fault with. Still it is certain that a bedroom of one's own is a great boon, especially to a dweller in hospitals, where the sleeping apartment offers the only chance of solitude. The authorities of this hospital are therefore gladly making preparations for building a new detached nurses' home, which will give each nurse a room to herself. They have been enabled to do this by a bequest from the estate of Mr. and Mrs. Pfeiffer of £3,000. The nurses are looking forward to the ceremony of stone-laying in a few weeks' time.

### AMERICAN RED CROSS NURSES IN CUBA.

Miss Clara Barton, the well-known President the Red Cross Nursing Society of America, has been commissioned by the *Christian Herald* to proceed to Cuba and investigate the sufferings caused by the rebellion. Miss Barton is guaranteed the sum of 10,000 dols. a month for expenses, so that she will be able to direct the relief organisation with a free hand.

### THE NURSES' EXPRESSION OF GRATITUDE.

The nurses of the Great Yarmouth Workhouse have sent a letter to the local press expressing their sense of the kindness they have experienced at the hands of Dr. Collier, and their regret that their action in asking the Guardians to defray their medical expenses should have been the cause of exposing him to a groundless attack. Some years ago Dr. Collier offered to attend the nursing staff without charge, an offer refused by the board. The action of the Guardians in this matter appears arbitrary; they refuse free medical attendance for their nursing staff, and yet expect their nurses to pay their doctor's bills.

### NURSING IN THE MIDLAND COUNTIES.

The annual general meeting of the Birmingham and Midland Counties Training Institution for Nurses disclosed a prosperous state of affairs. The staff, consisting of 73 trained nurses and 11 probationers, have been fully employed excepting for a short time in the autumn, and the nurses themselves have been most popular. The funds are in a flourishing condition, and bonuses both from the earnings of 1896 and from a special Jubilee gift have been distributed amongst the staff.

### SHORT ITEMS.

About £200 has been raised for the newly-formed Doncaster District Nursing Association. At a meeting held not long ago the new officers were appointed, and Mrs. Huntriss, who has taken the initiative in the movement, was appointed secretary.—The interest on the Diamond Jubilee Fund, amounting to £1,260, will be handed over by the Town Council of Greenock to the Medical Aid and Sick Nursing Society for the support of a Queen's Nurse.—A rise all round has been granted to the officials of Keighley Workhouse. Charge nurses now receiving £25 will have £28, with a yearly increment of £1 per annum until £32 is reached.—The rules for the nurses at Coventry Workhouse decree that the day nurses are to go on duty at eight a.m. and to remain until nine p.m., and all lights are to be out in the bed-rooms at half-past ten. Fourteen hours are to be allowed weekly for recreation, with half a day's leave once a fortnight. The date to be fixed by the superintendent nurse, having a due consideration to the state of the weather.—Mr. George Bolton has gived £200—the balance of the debt—to the Kingston Nurses' Home. The committee have had considerable anxiety the last year on account of the grave illness of the superintendent, Miss Scott, who eventually was obliged to resign. She was succeeded by Miss Penrose, who is fulfilling the duties satisfactorily.—The University of Buda Pesth has conferred the degree of Doctor on the Queen of Roumania.

### Lectures to Surgical Nurses.

By H. A. LATIMER, M.D. (Dunelm), M.R.C.S. of Eng., L.S.A. of London, Consulting Surgeon, Swansea Hospital; President of the Swansea Medical Society; Lecturer and Examiner of the St. John Ambulance Association, &c.

### XXVI.—CONCLUDING REMARKS ON THE TREATMENT AND CONSEQUENCES OF BED SORES—CONVULSIONS.

I WILL not assume that any formidable bed sores will form in patients under your charge, but you will often have people placed under your care who will have been badly nursed before you see them and in whom these ulcers will have formed. You must know, then, what to do in such an event. If the sores are covered with sloughs these must be removed before a healthy and healing surface can be obtained. The diseased spot must be at once isolated and relieved of pressure by air pillows, or carefully arranged cushions, and some stimulating poultice or preparation must be applied directly to the sore. A poultice formed of equal quantities of mashed carrots and bread crumbs is an excellent application, or a simple linseed meal one may be used. Whatever kind of poultice is put on the sore it should lie only on its open surface, and whenever it is changed the sore should be douched with an antiseptic lotion of some sort or other—say, 1 in 40 carbolic lotion, or a lotion made by dissolving as much boracic acid in hot water as the latter can take up. As soon as the sloughs have separated, the now raw surface is to be treated by stimulating and healing ointments or lotions. An excellent ointment for this purpose is made of eight grains of carbolic acid, half an ounce of resin ointment, and half an ounce of vaseline. As the granulations approach the surface and show that the ulcer may be healed over, ordinary zinc ointment is to be applied, and then the process of cure will be quickly completed. But as it will be very seldom that you will be without the guidance of a medical man when bad cases of bed sore have to be treated, I may assume that I have now said as much as is necessary on this part of my subject, and so may bring this lecture to a conclusion after saying a few words on the consequences which ensue from the formation of bed sores. You must not simply look upon these as painful spots, situated in positions which require great care on your part to shield from further extension, and as sources of pain and distress to their unfortunate possessors; they are all this, and more. Their mischief does not begin and end at the spots where they are situated, for they have far-reaching results in the system at large. You must regard them as so many suppurating wounds, through the medium of which infective micro-organisms may gain access to the blood. Thus their true importance must ever be borne in mind by you; and I would have you never weary in searching for, and anticipating their advent, in all cases where there is the least liability to their occurrence. I almost fear that I dealt too leniently with the subject in my introductory remarks about it, for I would not have it said that by any too considerate view of a nurse's duties I have implied that she would be held blameless if bedsores formed in a patient under her charge. There will always be a disposition on the part of doctors to blame nurses in such an event. As I have just said, bed sores are in the great majority of cases evidences of want of care or of skill on their part. All I meant then, and mean now, is to protect you against unjust accusations on this score. I must not be taken to mean that you are ever to regard these ulcers from any other point of view than the one which looks upon them as prejudicial alike to the welfare of the patient, and to the reputation of the nurse.

Many of the surgical affections of which I have been telling you in the preceding articles are complicated by convulsive movements in the body, but it is especially in diseases affecting the brain, spinal cord, and nerves, that the fact becomes of primary importance, for now convulsions become the characteristic manifestations of disturbed function and are veritable signs by which we can label and distinguish one disease from another. To make my meaning plain, I will adduce as an illustration of a convulsive movement, complicating a general disturbance in the body, the every-day occurrence of rigors, when suppurative fever is affecting the system. Here, owing probably to the circulation of a poisoned blood, which acts as an irritant on the nervous centres, an invalid is quite suddenly seized with an attack of violent shivering, so violent that his whole body shakes to and fro; and in young childhood the same irritant is likely to produce still more pronounced effects, not stopping at the shivering stage, but actually setting up fits with loss of consciousness. But in these and in other such cases due to irritation of the nerve centres by depraved blood, the convulsions are secondary in origin to disease elsewhere than in the nervous system; whereas I shall in my next proceed to describe them as arising from disease which affects that system from the start, and thus renders them a distinctive and distinguishing feature in the catalogue of diseases.

The terms "convulsion" and "fit" are interchangeable ones, and I see that a dictionary definition of them is "a violent and involuntary spasmodic contraction of the muscular parts of an animal body." They vary in degree and severity, and at different times, in the same subject, as I shall have to tell you when speaking of their occurrence in epilepsy: and they may be strictly localised to one set of muscles, or they may affect the body at large. The actual movements of muscles concerned in the spasms, or convulsions, which you may be watching, are secondary to an irritation conveyed to them by the nerves which bring currents of nerve force to them; they are responses to those irritations, just as the explosion of gunpowder is a response to the agent with which you have fired it. In both cases matter is lying inert, and assumes a state of activity on receipt of its appropriate irritant. Pathologists are apt to use this simile when they speak of convulsions being due to "explosions of nerve force." When the muscular movements are slight in amount it is customary to speak of them as "spasms," when more intense as "convulsions," but these terms are indicative only of degree and extent of movements, and in no way should be taken to indicate a different mode of irritation of muscles. As far as the movements of the muscles themselves are concerned the words are used in a peculiar sense, the fact of their being affected at all in this way being spoken of in a general sense as convulsions, whilst the word spasm is applied to the very movements of the muscles themselves, with a qualifying word as a prefix to denote the method of movement in them. When muscles are in receipt of violent irritation they contract, and they may either remain rigid and fixed in this shortened position, or may go through a series of sudden contractions and relaxations, following quickly the one upon the other. When they remain contracted for a while the public say they are affected by "cramp"; when they contract and relax alternately, they say the muscles are twitching. Under like conditions pathologists tell you that they are affected by "tonic" or "clonic" spasm.

### Wants and Workers.

CAN anyone inform Nurse Jarvis of a home or institution into which it would be possible to get admission for a crippled boy, aged about 13? His general health is good, and he can dress and feed himself, and is not of weak intellect; but, owing to neglect, has lost all powers of locomotion.—*Hitchin Workhouse.*

### Antiseptics for Nurses.

By a Medical Woman.

### IV.—COMPOSITION AND USES OF SOME OF THE MORE COMMON ANTISEPTICS.

### Carbolic Acid.

Some of the most generally used antiseptics are obtained from coal tar, a substance which was used for preserving wood long before it was so prepared as to give us such products as carbolic acid, creosote, creolin, izal, as well as various patented fluids and powders. Coal tar and its products have been in use as recognised antiseptics and disinfectants for over eighty years, and hitherto, though other antiseptics have appeared on the scene, they have been found wanting in some important respect, and carbolic has been reinstated as, though not perfect, the best antiseptic on the whole.

Coal tar and carbolic were used extensively on the Continent in the treatment of wounds long before they were so employed in this country. One of the earliest forms in which coal tar was used was as a paste, having the following ingredients: Ordinary plaster of commerce, 100 parts, and coal tar, 1 3 parts. The plaster was finely powdered in a mortar, then the coal tar added, and, finally, enough pure olive oil to make it into a suitable paste. This preparation attracted much notice and discussion at the time, and opinions as to its uses were very varied, some claiming for it true antiseptic properties, others maintaining that it was only a deodorant. Finally, a commission was appointed to investigate the properties of all the substances which claimed to be antiseptics, including the above powder, and that verdict was that, although a useful disinfectant for many substances, it was not suitable for wounds; it had a disagreeable odour, soiled linen, and required frequent renewal.

Two years later, in 1860, a French surgeon, named Lemaire, used coal tar, to which he added four parts of an alcoholic tincture of saponine; this formed an emulsion, and was used to wash the wound, and then "charpie" soaked in it was applied as a dressing. Lemaire considered this an active antiseptic containing three antiseptics—carbolic acid, alcohol, and saponine—which would arrest fermentation and act as a powerful disinfectant. Lemaire used carbolic acid experimentally, and published his results in 1863, which were that carbolic acid is more powerful than coal tar and is readily soluble in water, alcohol, or oil, but it is extremely volatile, and has a caustic effect on wounds. These last two properties were regarded as decided disadvantages, but Lemaire's discoveries excited great interest in his own country. In England, however, carbolic acid was only used by one or two surgeons, who failed to obtain good results, and consequently it fell into disuse, and it was reserved for Sir Joseph Lister to set forth the true principles of antiseptic surgery, and in connection with this to revive the use of carbolic acid.

Carbolic acid or phenol is obtained by distilling tar, and before it is purified it is a dark oily fluid with a strong tarry smell. In its purified state it is a white crystalline substance, but exposure to air turns it a pink colour, hence it is familiar to most of us as a reddish crystalline substance. It is not very soluble in water, only in the proportion of 1 part of carbolic in 15 of water, but it is more soluble in glycerine in the proportion of one part carbolic to two of glycerine. Sir Joseph Lister speaks very highly of this antiseptic, which has one special advantage over others, since it will so mix with the fatty matter on the surface of the skin that it will exercise its antiseptic properties even though the skin has not been previously cleansed with soap and water.

The ordinary hospital solutions in general use are of the strength of 1 part pure carbolic to 19 and 39 respectively of water, these forming the familiar 1 in 20 and 1 in 40 solutions. The private nurse will probably be supplied with Calvert's carbolic acid No. 4, and will have to dilute it to the required strength. The only preparations used in surgery are: (1) Carbolised oils, prepared by adding to olive oil pure carbolic in the proportion of 1 in 5, 1 in 10, 1 in 15, and 1 in 20, but these have disadvantages, and hence carbolised vaseline is substituted; (2) carbolised gauze, which is prepared by dipping fine, unbleached, previously washed muslin in a mixture of four parts each of common resin and paraffin and one part carbolic, the use of the resin being to retain the volatile carbolic acid, and prevent it from being washed out too quickly by the discharges, whilst the paraffin lessens the stickiness of the resin. After being thus prepared the muslin is dried, and must be kept in air-tight boxes. (3) Carbolic wool is prepared by saturating absorbent wool with carbolic acid to the extent of 5 per cent.

Carbolic soaps are practically valueless as antiseptics, and no nurses should rely on them, but should use ordinary soap and carbolic lotion.

Carbolic tooth powder is an excellent preparation for those who do not dislike the taste, but unfortunately the valuable ingredient, the carbolic, volatilises long before the powder is finished as a rule. The only patent preparation of pure carbolic is Rademann's, which consists of carbolic and pure boric acid.

### Creosote, Creolin, Izal.

After carbolic or phenol has been obtained from coal-tar, certain so-called "waste products" are left, and, lately, creosote, creosol, and creolin have been obtained from them.

Perfectly pure creosote usually looks like water, though sometimes it may have a yellowish tinge. It has a strong aromatic odour and a burning taste. It is only very sparingly soluble in water, but freely so in alcohol. It is said to be as good an antiseptic as crude carbolic acid, and not to have so caustic an effect on the skin. However, creosote is not used in surgery, as its strength and composition are uncertain, and carbolic is more satisfactory. It is very useful for inhalations and for decayed teeth.

The other kindred preparation of gas-tar—creolin—is purer than creosote. It is brownish-black syrupy fluid, with a pleasant aromatic smell, and is like gas-tar both in this and in appearance. It is not soluble in water, but can be used as an emulsion of 1-3 per cent. strength for washing wounds and soaking dressings; but this emulsion must be prepared freshly, just before each dressing, otherwise it becomes a dirty brown liquid if it stands long. Creolin is also prepared as an ointment in ½-2 per cent. strength. It is one of the most powerful of the non-poisonous antiseptics, in this respect belonging to the same group as boracic, eucalyptus oil, and salicylic acid, and is perfectly free from irritating effects on the wound. It is strongly recommended by some surgeons, and is considered by Erichsen to be "a most efficient and unirritating antiseptic." That most familiar disinfectant, "Jeyes' Fluid," is a preparation of creosote with resin and soda. Another preparation is a patent disinfectant known as "Evrits' Fluid."

The remaining preparation, Izal, is obtained when coke is carbonised in close stoves. It is a reddish-brown fluid, which forms a milky emulsion with water. It has been employed as an antiseptic in surgery, but has not come into very general use.

### NOTTINGHAM AND NOTTS NURSING ASSOCIATION.

We hear from the lady superintendent of the above that the subscriptions asked for are on behalf of the district nurses. The private nursing staff is self-supporting, and their surplus earnings is spent entirely for their benefit.

### Nursing in Paris Hospitals.

### A.—LAY NURSES.

### V.—Wages and Pensions.

In contrast to almost all employments where the two sexes are at work side by side, the woman nurse in Paris receives the same pay and allowances as the man in the same grade. The number of the grades above probationers is now five, viz.: (1) Assistant nurses (infirmiers and infirmières); (2) staff nurses (premiers infirmiers and premières infirmières); (3) assistants (suppléants and suppléantes); (4) assistant superintendents and matrons (sous surveillants and sous surveillantes); and (5) superintendents and matrons (surveillants and surveillantes). The wages of these five classes are as follows, as fixed by the administrative circular of June 25th, 1897, in accordance with the vote of the Municipal Council in December, 1896. As will be seen, the first, second, and fifth grades are again subdivided into two classes, and the wages of the household staff are appended, being, in fact, identical with that of the lowest grade of actual nurses :—

| | | Lodged. | Not lodged. |
|---|---|---|---|
| Superintendents & matrons, | 1st class | 884 francs. | 2,284 francs. |
| " " | 2nd " | 784 " | 2,184 " |
| Assistant ditto ... ... | 1st " | 684 " | 1,984 " |
| " ... ... | 2nd " | 584 " | 1,884 " |
| Assistants ... ... ... | — | 488 " | 1,588 " |
| Staff nurses ... ... | — | 444 " | — |
| Assistant nurses ... ... | 1st " | 423 " | — |
| " ... ... | 2nd " | 393 " | — |
| Porters and housemaids | 1st " | 423 " | — |
| " " | 2nd " | 393 " | — |

Although the sexes are on an equality while in active service, for some inexplicable reason the pensions accorded after retirement give the usual advantage to the male recipient. The minimum qualification for a pension is a service of 15 years, ending in one of the three upper grades, exclusive of cases of serious disability incurred by reason of hospital work. The scale of pensions ranges as follows: For assistants, 230 francs a year for men and 195 francs for women after 15 years' service, with proportional addition for each year of service, to 550 francs for men and 480 francs for women after 30 years of service; for assistant superintendents and matrons the pensions range from 265 francs for men and 215 francs for women after 15 years' service, to 640 francs for men and 540 francs for women after 30 years' service; for superintendents and matrons the pensions range from 310 francs for men and 260 francs for women after 15 years of service, to 750 francs for men and 650 francs for women after 30 years of service. All the pensions are divided into pensions proper and extraordinary allowances, the figures given being the united totals of the two. This so-called extraordinary allowance was something which came into practice about half a century ago, but has now become so ordinary and so inevitable as to make the division a mere fiction. Although the employés in the offices of the Assistance Publique have five per cent. deducted from their pay for pensions, no such deduction is made in the case of the nursing staff.

There may possibly be hereafter an unforeseen increase of the expenses for pensions in a peculiar way. Although the hospital chiefs are almost unanimous in commending the working of the new rules concerning engagements, nevertheless, one of the keenest of them told me that he would prophesy a serious cost to the municipality in the matter of retirements. Henceforth every retiring nurse will claim any serious illness or permanent debility, especially anything resulting from epidemics or contagion, as a malady contracted in course of duty. This will entail claims to many increases of pensions not otherwise earned. Henceforth after the double guarantee of two medical examinations, the nurses will be able to say "we were hale and sound when we entered the service." Even where by their own folly or vice they contract debility, it will always be said to have been brought on by the work.

This opinion is, however, at variance with that of another hospital director, who says that the nurses have no reason to take any disease from their work if they observe the rules and precautions ordered. Hence the administration will be in a position to deny all claims for such future debility as a result of hospital work.

The recent augmentation of the nurses wage scale leads me to refer to the constant expansion of these expenses since the Paris Assistance Publique was organised on January 10th, 1849. The wages were then left for the nursing staff as found in force (being those devised by the previous organisation in 1837) as follows: 1st class sub-employes (alias surveillantes or superintendents), 300 francs a year; 2nd class sub-employes (alias sous surveillantes or assistant superintendents), 210 francs a year; men nurses (these not sub-divided), 180 francs a year; women nurses, 150 francs a year; porters, 150 francs a year; housemaids, 120 francs a year. Of course these grades were all lodged and fed. They also had occasionally special increases for long and faithful services.

In 1853 the scale was increased to 360 francs for first-class sub-employés, and 250 francs for the second-class, the rest remaining unaltered.

In 1861 came another and larger increase, with a complete reorganisation. The first-class sub-employés were given 360 francs per annum, with an annual increase of 26 francs to 500 francs a year. The second-class had 320 francs, with an increase of 12 francs yearly to 380 francs. The first-class assistants (suppléants), now first instituted, were given 252 francs, with an annual increase of 12 francs to 300 francs a year. The assistant nurses and servants were given 180 francs, with an annual increase of 18 francs to 252 francs a year. It was this scale which first established equality of the sexes in all grades.

In 1875 the lowest grade was ordered to have the maximum wages after two years of service.

Since the lay campaign began in 1878 there have been no less than five increases of salary—in 1879, in 1882, in 1894, and the present scale in 1897. These changes previous to the 1897 one, already detailed, have been as follows:—

| Grade. | 1879. Francs. | 1880. Francs. | 1882. Francs. | 1894. Francs. |
|---|---|---|---|---|
| Superintendents, 1st class | ... 600 | ... 680 | ... 800 | ... 800 |
| Ditto, 2nd class ... | ... 510 | ... 540 | ... 700 | ... 700 |
| Assistant ditto, 1st class | ... 450 | ... 480 | ... 600 | ... 600 |
| Ditto, 2nd class ... | ... 400 | ... 420 | ... 500 | ... 500 |
| Assistants... ... ... | ... 330 | ... 360 | ... 400 | ... 425 |
| Staff nurses ... ... | ... 300 | ... 330 | ... 360 | ... 400 |
| Assistant nurses, 1st grade | ... 270 | ... 300 | ... 330 | ... 380 |
| Ditto, 2nd grade ... | ... 240 | ... 270 | ... 300 | ... 350 |
| Ditto, 3rd grade ... | ... 180 | ... 240 | abolished | |

Under the new rule, certificated nurses in lower grades have 3 francs a month extra while waiting for promotion, and M. Peyron has decided that henceforth all promotions must be made from the certificated claimants. I have already given the remuneration of the probationers. The certificated nurses have also frequent opportunities of going into the country for promotion, the laicisation in various towns making many openings. Promotions are often made on the recommendation of doctors who find a nurse especially apt, but these promotions have to be subject to the rules governing other promotions.

There seems no prospect that there will be any more permanency in the future in the wage scale than in the past. This very year the Municipal Council has voted an increase of 120,000 francs for the nursing staff, which sum has not yet been allocated. The present grievance of the employés is inequality in the pension allowances, especially in the matter of work in the asylums under the Department of the Seine not being counted as part of the term of service. There is always a tangle between the Department of the Seine and the municipality of Paris, and it is only natural that the nurses should be included in the meshes. EDMUND R. SPEARMAN.

### Diet for the Sick Room.

### FOUR TRAYS FOR INVALIDS.

We all know that when once influenza patients begin to convalesce their recovery is greatly aided by the amount and quality of the food they are able to take. As this malady is very prevalent just now, I will give a few hints that I hope will be useful in this respect.

I am well aware of the difficulties there are in many households to get the invalid's meals dainty and appetising; but the nurse can always control the tray, seeing that the cloth is spotless, the little cruets bright, cups and saucers not too large, and putting a few flowers to give colour. When possible, serve the chief thing on a separate dish, and avoid putting everything in the way of meat, vegetables, sauce, and gravy on to one plate. Patients will often eat more if they can help themselves, and there is not any chance of the second help getting cold if the meat and vegetables are kept on a hot-water dish and covered over.

### The Breakfast Tray.

Coffee or tea.
Sole, with tomato purée.
Brown bread and butter. Crisp toast.
Jaffa orange.

Serve the tea or coffee in a small tea or coffee pot, not a cup; if the latter, boiled milk should be in a separate jug. The toast can be put in a small rack or cut in sippets and piled on a plate. Do not peel the oranges unless requested to; if not very ill, patients often like to do it for themselves. To cook the sole, first wash it in salted water, trim off the fins and head, then wipe it with a clean cloth, place it on a buttered tin and sprinkle with a tablespoonful of lemon juice, a little salt and pepper, cover it with a buttered paper, and cook it in a moderate oven for ten or fifteen minutes according to size. When white and firm dish it up, and pour the following sauce round it: Cut up two tomatoes and cook them with half an ounce of butter, a little pepper and salt, and a pinch of castor sugar; rub it through a strainer or sieve, make it quite hot again, and use as directed.

### Dinner Tray.

Bouillon.
Fillets of beef.
Potato ribbons. French beans.
Compôte of rhubarb.
Sponge fingers.

Serve the bouillon in a small cup, with a lid to it; the fillets on a hot water dish with a nice gravy round them; the French beans and potatoes, if possible, in a vegetable dish with two divisions. Remove everything belonging to this course before serving the sweet. Instead of a slice of bread a nice light dinner roll is often liked. As to beverage, soda water or a lemon squash are general favourites. Milk is not necessary with an ordinary meal like the above; it is too heavy.

Fillets of Beef.—Cut two or three slices about three-quarters of an inch thick from a nice fillet, trim them, and lard them with thin strips of fat bacon. Place them on a buttered tin and cover them with a buttered paper. Cook them in a hot oven for four minutes, then take off the paper and continue cooking for a few minutes longer till the lardons are crisp; dish up on a hot-water dish, pour a nice piquant sauce round, put some small pieces of butter that have been mixed with a little lemon juice and chopped parsley on the fillets, and serve very hot.

### Tea Tray.

Chocolate.
Sugar, milk, or cream.
Cress sandwiches.
Tea rusks with apricot jam.

Use Vienna bread for the sandwiches, have the butter of the freshest, and shake all the water from the cress after washing it. Dish them up on a dish paper, overlapping each other like cutlets. The rusks are nice if very thinly spread with the jam.

### Supper Tray.

A French omelet.
Crisp toast.
Prune jelly. Water biscuits.

French Omelet.—Break two fresh eggs into a basin and mix them with two tablespoonfuls of cream or milk, add a little pepper, salt, and chopped parsley, that has been chopped very finely, then washed and wrung dry in the corner of a clean cloth, melt one ounce of butter in a small omelet pan, and when it is hot pour in the mixture. Stir it about with a wooden spoon. When it begins to set form it into half-moon shape; cook it for a minute or two longer till it is brown underneath, but not hard; loosen the sides with a knife, and turn it over on to a warm dish, and serve at once.

Prune Jelly.—Cook a pound of the best prunes till they are quite soft, strain them and save the liquor. Take out the stones of the fruit and rub it through a wire sieve. Make the liquor warm and melt in it four or five sheets of leaf gelatine and three ounces of lump sugar. Strain this on to the pulp that has been passed through the sieve, mix well together, then put it into some fancy mould; when set dip the mould in warm water, and turn out the jelly on to a glass dish. Whipped cream or plain custard round is a great improvement.

### Death in Our Ranks.

We regret to have to record the death of Nurse Emily Mary Dunn, a very promising probationer nurse at the Victoria Infirmary, Northwich. After six days' illness, she succumbed to an attack of pleuro-pneumonia. Miss Dunn was a native of Church Stretton, in Shropshire.

### "THE HOSPITAL" CONVALESCENT FUND.

We have much pleasure in acknowledging the receipt 2s. 6d. from Nurse Dinwoodie for the above.

### Miss Mary Kingsley at the Royal British Nurses' Association.

THE Royal British Nurses' Association is to be congratulated on having secured Miss Kingsley, the famous African explorer, to lecture on the 8th inst. The next time they arrange such a treat for their members it would only be kind if they made it a public affair. Mrs. Dacre Craven, who had left a sick bed to be present, took the chair, and introduced the lecturer in a few words. The lecture was illustrated by lantern slides.

Miss Kingsley began by giving her views on how nursing should be organised on the West Coast of Africa, as, she said, good intentions did not necessarily lead to success, and her experience might be useful. She thinks that, taking all things into consideration, a staff of hospital ships lying off the coast, with branch hospitals on shore, each officered with trained nurses and orderlies, the most practical way in which to deal with the nursing question of the region. Of course, she remarked, there are difficulties; there is, for instance, bilge water. This is a topic of fascinating interest to people who understand the question. It is certainly the prince of smells. Once on a vessel she perceived a decidedly objectionable smell, and on mentioning it she received for reply, "It is only our bilge." In the morning it was discovered that an elephant in an advanced state of decomposition had somehow or other got jammed against the side of the ship. The smell was so awful when the carcase was being cleared away, that she suggested that they should stir up the bilge and let the two smells annihilate one another. "No," was the horrified response; "you don't know our bilge when its back is up. It would stretch you in South Africa." Then the motion of the hulk has to be taken into consideration. The Atlantic Ocean is not rough unless there is a tornado blowing, but, as a rule, there are tornadoes twice a day. Not that one gets two tornadoes every day, but that is the average. The tornado certainly relieves the monotony of life on board of a hospital ship; occasionally it drifts it out to sea, but that is not alarming, as the people on shore would be sure to send out to seek it, especially with white patients and lady nurses on board. If the Atlantic is not rough unless there is a tornado blowing, neither is it smooth. Nevertheless, all the disadvantages of a hospital ship do not outweigh the cleanliness and healthiness to be obtained on board. If cruisers and not hulks were used the advantage would be greater; or, as an alternative, if the mail steamers had a hospital cabin with a trained nurse in charge (taking the nurses attached to the inland hospitals in turn) the benefits to all would be greater still.

Miss Kingsley next proceeded to give some practical suggestions. It is true that malarial fever repays nursing, but the white men are scattered, and the transport bad, and a long hammock journey would certainly do the patient more harm than good in the majority of cases. So that the best nursing arrangements could only touch the fringe of the need. The principal requirement of the pioneers is good cooking, which every explorer should understand. A man gets a little out of sorts, and has a touch of fever: his appetite rebels against tinned meat, and consequently he grows weaker, and the fever gets a stronger grip. His appetite gets worse, and the fever stronger, until he falls ill, and perhaps dies. There is plenty of fish in the African rivers, plenty of vegetables in the forest, and the native women are often good cooks, but the white men do not come much in contact with them. Then an explorer should take Dr. Cross' "Hospital Notes for Expeditions," and learn something of the dangers of drugs. Amateur doctors are all bad, but the male variety is worse than the female. The former may select a remedy by chance from a number of drugs, but the latter is generally faithful to one patent medicine. As increasing numbers of Englishmen go to Africa, it is a reproach that nursing is not systematically arranged for them. The Germans have their trained nurses, the French trained orderlies, and the Portuguese their sisters of mercy. The reason that the English are behindhand is that the colonies cannot afford the heavy expenses which trained nursing entails. This ought surely to be borne by the Imperial Exchequer. Two staffs of nurses would be required—one on duty and one at home, and a few to spare. Plenty of holiday is necessary. It is not desirable that the death-rate for nurses should be as high as it is for Government officers or for traders, which is 35 per cent. in some places, 52 per cent. in others, and 75 per cent. in another. White women show greater immunity to fever than white men. This is not because the women live a more guarded life, for the bush-exploring and exposed life is healthier than the rabbit-in-a-hutch existence which many Europeans are condemned to.

Miss Kingsley has a great admiration for some of the white women working in Africa. She gave a graphic description of turning up at a Roman Catholic Mission and finding the Mother in the midst of her dispensary work. "Just hold his legs whilst I scrape his wounds" was her greeting, an order which she promptly obeyed, and rendered what help she could until the work was finished. "You'll do," said the Mother. "Come and have a cup of tea." Then she learnt that she was supposed to have been the Danish assistant that had been expected for the last eight months. This "Mother" always slept with her door open, in case anyone should want her in the night. As a matter of fact, someone always did want her in the night.

It was most eerie to watch the patch of white moonlight outside, and see a silent, dark figure steal through the open door, as if its sole purpose was to avoid disturbing the lightest slumbers, and which would come and crouch on the floor at the bedside, and then gave vent to the most blood-curdling howl. The programme on these occasions was always the same: (*a*) Find the matches; (*b*) light the lantern; (*c*) if the child was ill give a dose out of No. 1 bottle; (*d*) if the mother (these figures were generally mothers and their babies), a dose out of No. 2 bottle (there were only two medicines for night work, one for the adults and one for the children); (*e*) pilot the patients to the quarters provided for them at the other side of the yard; (*f*) return to bed and hope that they would stay where they had been left. These proceedings were varied one night by a goat being the visitor, and a scene of great confusion ensued which nearly wrecked the mission buildings.

The second instance was even more exciting. She found a steam launch fast on a sandbank with only an engineer in charge, and he was ill and "mixed up with the machinery." As his finder knew nothing about machinery, and less about men, she had some difficulty in disentangling the one from the other. Then she set to work to pole the boat free, an occupation that kept her fully employed for hours, but which was varied by having periodically to disentangle her patient from the machinery, to which he gravitated immediately her back was turned. After succeeding in floating her craft and mooring it alongside the bank she prepared to take her well-earned rest, feeling very proud of herself at having ensconced her patient under an impromptu mosquito net she had rigged up for him. Her conceit was soon crushed out of her, for at nightfall the launch was invaded by legions of snakes. In a pandemonium of hurry and confusion she pushed out into the river once more. Some days later, when the settlement was reached, and her patient was recovering, he informed their hosts "that he had told her what would happen if she moored at the river bank." Considering, however, that she could not understand his language, and thought him delirious, it was hardly to be wondered she did not follow his advice.

This lecture is to be published by the author.

### For Reading to the Sick.

### THE ROAD OF SORROWS.

### Verses.

WELL may we mourn our dull, cold heart and eye,
That up the mount of Glorious Sacrifice
Sees such a little way! Yet kneel we nigh,
Turn not away; let prayer in gloom arise.
—*Keble.*

With all His sufferings full in view
And woes to us unknown,
Forth to the task His Spirit flew;
'Twas love that urged Him on. —*Cowper.*

Then like a long-forgotten strain
Comes sweeping o'er the heart forlorn,
What sunshine hours had taught in vain,
Of Jesus suffering pain and scorn.
As in all lowly hearts He suffers still,
While we triumphant ride and have the world at will.
—*Keble.*

Well I know thy trouble,
O My servant true;
Thou art very weary,
I was weary, too;
But that toil shall make Thee,
Some day all Mine own,
And the end of sorrow
Shall be near My Throne.

Here I rest for ever viewing
Mercy poured in streams of Blood;
Precious drops, my soul bedewing,
Plead and claim my peace with God.
Truly blessed is the station
Low before his Cross to lie,
Whilst I see Divine compassion
Beaming in His languid eye.

Could'st thou not suffer then, one hour—or two?
If He should call thee from thy cross to-day,
Saying, "It is finished! That hard cross of thine
From which thou prayest for deliverance,"—
Thinkest thou not some passion of regret
Would overcome thee? Thou would'st say, "So soon?
Let me go back and suffer yet awhile
More patiently! I have not yet praised God."
And He might answer to thee, "Never more,
All pain is done with!" —*H. Hamilton King.*

### Reading.

Christians are fond of dwelling on the sufferings of their Master, and the Way of the Cross is a devotion that brings home to us very plainly what He endured for us. We do not recall these sufferings to compassionate them. They are over for ever, and the sacred Manhood of Jesus can suffer no more. But we recall them in order to deepen our hatred of sin. Each bloodstained footprint was caused by sin. As we think, then, of all the manifold insults and pains inflicted on Jesus, we ought to learn to hate the sin which caused them, and bitterly to repent of our own share in causing them. But we dwell on His sufferings for yet another reason. They are the proof and evidence of His love for us. By the very redundance of His pains He calls to us. He seems to claim our love by the multitude of sorrows He has borne for us. As we watch that pale Form staggering along beneath the heavy Cross, or see it outstretched upon the Cross and lifted up, we see that Jesus loves us and calls us by His suffering. Can I be so ungrateful, so heartless, that I will not thank Him for His love?

### Appointments.

### MATRONS.

LEWISHAM UNION INFIRMARY.—Miss Hetty E. A. Dixon was elected Matron of the Greenwich Infirmary on March 3rd. Miss Dixon was trained at King's College Hospital, and remained there from March, 1892, until February, 1896. She was appointed ward sister at this infirmary in February, 1896, and was promoted in July, 1896, to be assistant matron, which post she has held until the present time.

CHARNWOOD FOREST CONVALESCENT HOME, LEICESTERSHIRE.—Miss Eleanor Waldron was on March 2nd appointed Matron of this hospital. She was trained and afterwards became ward sister at Leicester Infirmary. Miss Waldron was subsequently matron at Chesterfield.

### Minor Appointments.

THE INFIRMARY, GRESSENHALL UNION, EAST DEREHAM, NORFOLK.—Miss Doughty was elected Superintendent Nurse on January 11th, of the above infirmary. She was trained at the Norfolk and Norwich Hospital, where she remained five years. She was afterwards charge-nurse at Chichester, and at the Fever Hospital, Southend-on-Sea, and nurse matron of the infirmary wing, St. Pancras Workhouse.

ROYAL INFIRMARY, WINDSOR.—Miss Edith Dring, who was trained and afterwards staff nurse at St. George's Hospital, London, was appointed Charge Nurse on March 3rd. Miss Dring has been engaged in private nursing for the last two years.

GREENWICH UNION INFIRMARY.—The new Head Nurse appointed on the 3rd inst. is Miss Bullock, who was trained at the Infirmary, Salop. She has previously held appointments as nurse at the Herne Bay School, the Infirmary, Salop, and Norwood College Hospital.

FORSTER GREEN HOSPITAL FOR DISEASES OF THE CHEST, BELFAST.—Miss Mary Mackenzie has been appointed Sister of the above. She was trained at the Hospital for Consumption and Diseases of the Chest, Brompton, and at St. Thomas's Hospital.

NOTTINGHAM UNION HOSPITAL.—Miss E. F. Dwight, who has hitherto had charge of the male wards of the above hospital, has been elected Nurse Superintendent. She was trained under the Southern Workhouse Association.

### Notes and Queries.

*Expenses of a Cottage Hospital.*

(194) What would be the yearly expenses of a cottage hospital of four beds worked by two nurses on very economical rates? Could it be done for £270?—*C. A. Cooper.*

The question is too wide and complicated to be settled in a few words. Burdett's "Cottage Hospitals" contains all the information which could be required by anyone who undertakes to found or to manage a cottage hospital. Price 10s 6d., from the Scientific Press, 28 & 29, Southampton Street, Strand.

*Crushed Malt.*

(195) Could you kindly tell me where this, which is recommended in a recipe for consumptive patients' diet, may be had?—*Nurse Gage.*

The malt for infusion can be procured at any good family grocer's.

*Daily Nurses.*

(196) Would three or four thoroughly trained nurses living together be likely to find remunerative daily work as private nurses?—*Nurse W.*

So much depends on the nurses and the way they manage. The Secretary, the Victoria Commemoration Club, 28 and 29, Southampton Street, Strand, could possibly give some useful information.

*Brighton.*

(197) Can you kindly tell me of a cheap convalescent home at Brighton? I am a nurse recovering from a bad attack of pleurisy.—*J. O. G.*

The London and Brighton Female Convalescent Home, Crescent House, Marine Parade, Brighton. Apply Lady President, 2, Eaton Place, Brighton. Terms 8s. to 10s. 6d.; reduced railway fares. Yours appears a suitable case for help from the Hospital Convalescent Fund.

*Too Old?*

(198) I am 43 years of age, and have been nursing for some years. I wish to become a charge nurse, and cannot do so until I have a certificate. Am I too old to train?—*Eva.*

Of course you are not too old to train, but is it worth while beginning to do so at your age? If you think so, why not consult your own matron? Many matrons dislike probationers who are over 30, and who have served in other institutions.

*Books and Training.*

M. T. must send name and address before we can reply to her query.

### ANSWERS REQUESTED.

*Sheets.*

(199) I have charge of a ward containing 29 beds, four of my patients being "dirty cases." I am allowed 120 sheets and 70 draw sheets, half being in use and half at the wash. As the linen only goes to the wash once a week, I find I have not enough, and should like to know how many are usually allowed under similar circumstances.—*Station.*

